# Paradoxical Insomnia in a Frustrated Patient Treated With Hypnotics for Ten Years

**DOI:** 10.7759/cureus.16234

**Published:** 2021-07-07

**Authors:** Waiz Wasey, Sharefi Saleh, Kristia Abernathy, Amit Sapra, Priyanka Bhandari

**Affiliations:** 1 Family and Community Medicine, Southern Illinois University School of Medicine, Springfield, USA; 2 Family Medicine, Ruth Temple Clinic, Los Angeles, USA

**Keywords:** insomnia, paradoxical insomnia, hypnotics, sleep disturbance, polysomnography, sleep medicine, primary care

## Abstract

Insomnia remains a common complaint for which patients present to their primary care providers. The reflex response by most primary care providers to treat insomnia is by prescribing hypnotics. The most commonly prescribed hypnotic is a sedative, such as a benzodiazepine or a benzodiazepine receptor agonist (BRZA). Paradoxical insomnia is a subtype of insomnia described as a complaint of severe insomnia disproportional to the presence of objective sleep disturbance or daytime impairment. Previously known as sleep-state misperception, this subtype of insomnia is not well known among the primary care community. We present a case of a 60-year-old female who had been prescribed multiple hypnotics for over 10 years and presented to our sleep clinic frustrated due to failure of treatment with each medicine. She was eventually diagnosed with paradoxical insomnia after an evaluation of her sleep parameters. This was effectively treated with cognitive-behavioral therapy. This case report aims to raise awareness of this subtype of insomnia in patients at the primary care level and to help minimize the use of hypnotics.

## Introduction

The prevalence of insomnia is around 10-30% worldwide, more common in older adults, female sex and in patients with mental health issues [1]. Insomnia is the inability to fall asleep or stay asleep despite adequate conditions. There are various subtypes of insomnia. A specific one we are presenting is called paradoxical insomnia, which exists in 5% of patients with insomnia. It is a complaint of severe insomnia disproportional to the presence of objective sleep disturbance or daytime impairment [2]. We present a case of a 60-year-old female who had been prescribed hypnotics for over 10 years and was found to have paradoxical insomnia following multiple polysomnographic evaluations. 

## Case presentation

A 60-year-old female with a history of bipolar 1 disorder, anxiety, hypertension, and chronic insomnia was seen in the sleep clinic for management of insomnia. She had struggled with not being able to sleep since childhood but reported it being worse in the last 8-10 years. She mentioned that there were days when she could not fall asleep for 72 hours and ended up in the emergency room (ER). She suffered from anxiety and worries about various aspects of her life. She believed that the anxiety made it difficult for her to fall asleep. In the last 10 years, she had been prescribed a battery of hypnotics that included but were not limited to Melatonin, Ambien, Seroquel, Doxepin, Prazosin and Gabapentin. She was responsive to these hypnotics initially but with time developed tolerance. She also reported unpleasant side effects from their use. She mentioned that it took her a long time to fall asleep, and she always woke up within a few hours. Additional sleep history included no snoring or witnessed apnea. No history of parasomnias such as sleepwalking or talking. She did have mild symptoms of restless leg syndrome. 

On examination she did have a narrow airway with a Mallapati score of 4 and scalloping of the tongue was noted. She also scored 0 on the Epworth Sleepiness Scale screening (Table 1).

**Table 1:** Result of Patient's Epworth Sleepiness Scale Screening

Chances of dozing off during	Score
Sitting and reading	0
Watching TV	0
Sitting inactive in a public place	0
Being a passenger in a motor vehicle for an hour or more	0
Lying down in the afternoon	0
Sitting and talking to someone	0
Sitting quietly after lunch	0
Stopped for a few minutes in traffic while driving	0
Total	0

At the time of evaluation in the clinic, the patient was on Hydrocodone-5, Seroquel and Ambien. She still reported an inability to fall asleep with these medicines. Surprisingly she had no daytime impairment as reported by the Epworth score screening, and denied needing any daytime naps.

**Figure 1:**
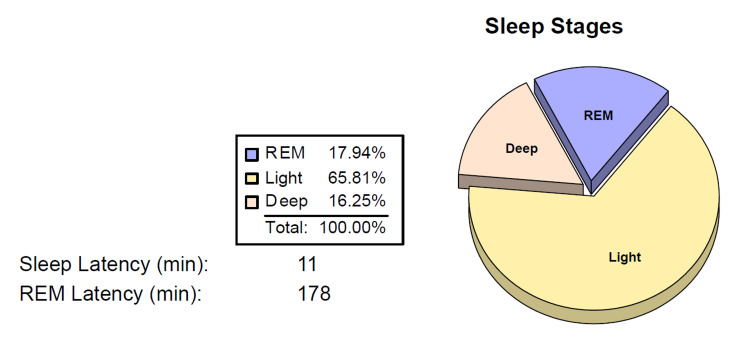
Sleep Stages on Home Sleep Study (March 2020)

A home sleep study was performed as an initial evaluation. The choice for a home sleep study instead of in the lab was done to make it comfortable for the patient to sleep in her own environment and avoid the first-night effect in the lab. The results of her sleep parameters by the home sleep study (March 2020) are presented in Table 2 and Figure 1.

**Table 2:** Patient's Home Sleep Study (March 2020) REM: rapid eye movement.

Sleep parameter	Value
Total sleep time	5 hrs, 54 mins
% of REM sleep	17.9%
Sleep onset	11 mins

While discussing the sleep study results, the patient reported not sleeping during the study. WatchPAT device was used for the home sleep study evaluation which utilizes peripheral arterial tonometry to detect sleep, sleep stages and any sleep apnea events. On a follow-up visit, the patient’s Epworth score was still 0. The patient was reassured that she did sleep well during the study and most likely had paradoxical insomnia based on the findings. During a follow-up after three months, the patient still believed she was not sleeping and did not think the home sleep study was accurate. 

**Figure 2:**
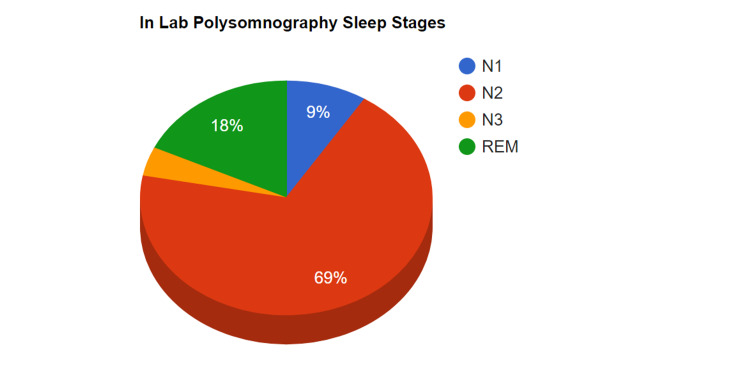
In-Lab Polysomnography Results With Sleep Stages (March 2021)

After detailed discussions, in-lab polysomnography was performed to evaluate stages of sleep in this patient utilizing electroencephalography (EEG). The findings from the polysomnography (March 2021) are displayed in Table 3 and Figure 2.

**Table 3:** In-Lab Polysomnography Results With Sleep Stages (March 2021) REM: rapid eye movement.

Sleep Parameter	Value	Misc
Total sleep time	325 mins/5 hrs 25 mins	Sleep efficiency 86%
Sleep onset	17 mins	
Stage N1	9.1%	
Stage N2	69.6%	
Stage N3	3.7%	
Stage REM	17.7%	

As evident from both studies, the total sleep time was similar and so was the REM percentage of sleep. Also the sleep latency, i.e., time taken to fall asleep, was less than 15 mins for both studies. The patient was reassured that two different evaluations done with different methodologies, a year apart, yielded the same results and that the patient was indeed sleeping five to six hours.

After educating the patient on her polysomnography results along with cognitive behavioral therapy, on follow-up visits in three months, she reported perceiving sleep for five to six hours. During this period, she was weaned off of Ambien as well. Her anxiety with regard to sleep also decreased significantly. Her Epworth sleepiness score remained unchanged.

## Discussion

Paradoxical insomnia, formerly known as sleep state misperception, is a subtype of insomnia that is characterized by self-reporting of insufficient sleep quantity and/or quality with little objective evidence from a polysomnography [3,4]. Patients with paradoxical insomnia complain of sleeping for a few hours, or not sleeping at all. Our patient presented with a similar complaint to multiple primary care and psychiatric providers before being seen in the sleep clinic. Since these patients have a heightened awareness of environmental stimuli and mental activity as they try to sleep, they perceive very little or no sleep. In reality, these patients are obtaining an adequate amount of sleep. 

The etiology of paradoxical insomnia is not well understood, but some hypotheses suggest the role of personality traits [5]. A positive psychiatric history is often associated with this subtype of insomnia. It has been found to be comorbid with schizophrenia, depression, anxiety, bipolar disorder and alcohol dependency. Our patient had a history of anxiety and bipolar disorder. 

Epworth sleepiness scale (Table 4) is a subjective screening tool to measure daytime impairment from inadequate sleep [6]. It consists of subjectively measuring chances of dosing off in eight different scenarios and is scored from 0 to 24. A score greater than 10 is significant for daytime impairment as a result of underlying sleep disorder. Patients with paradoxical insomnia usually score <5. Our patient consistently scored 0, which meant that she was getting adequate sleep during night time which did not affect her functioning during the day.

**Table 4:** Epworth Sleepiness Scale

Chances of dozing off during	No chance	Mild chance	Mod chance	High chance
Sitting and reading	0	1	2	3
Watching TV	0	1	2	3
Sitting inactive in a public place	0	1	2	3
Being a passenger in a motor vehicle for an hour or more	0	1	2	3
Lying down in the afternoon	0	1	2	3
Sitting and talking to someone	0	1	2	3
Sitting quietly after lunch	0	1	2	3
Stopped for a few minutes in traffic while driving	0	1	2	3
Total				

Polysomnography is not required to diagnose paradoxical insomnia, however, it can be ordered to rule out sleep-disordered breathing such as obstructive sleep apnea (OSA). In our case, polysomnography was done to rule out sleep apnea and periodic limb movements. Since they were ordered, we used the sleep stages from the studies to reassure the patient that she was sleeping adequately. Our patient had a perceived loss of sleep with no objective evidence from the multiple polysomnography evaluations. There was also no evidence of daytime impairment since her Epworth score was 0 and she was not requiring any daytime naps to feel restored.

About 5% of patients with insomnia have paradoxical insomnia. Due to its low prevalence, this subtype of insomnia is often overlooked and treated with hypnotics based solely on a patient's complaint. Our patient was treated with multiple hypnotics for over 10 years by primary care physicians and psychiatrists. She reported limited response to these hypnotics and more unpleasant side effects from the medications. There are no clear guidelines on the treatment of paradoxical insomnia. Treatment is categorized as pharmacological and non-pharmacological. When treated with medicines, benzodiazepine or benzodiazepine receptor agonists have shown effectiveness in patients to identify sleep [7], but patients develop tolerance soon. This happened with our patient when she used Ambien. Other treatment options include risperidone and olanzapine.

The non-pharmacological treatment for paradoxical insomnia includes cognitive behavioral therapy for insomnia along with explaining the discrepancy of self sleep perception and the objective findings on the polysomnography [2]. This is the method we adopted for our patients. Since pharmacological treatment options come with unwanted side effects, psychoeducation provides a safer treatment option. We had weaned our patient off of Ambien and solely resorted to psychoeducation. Her perception of sleep improved with sessions. As we do not have enough research in this area, more specific evidence-based guidelines are needed for the treatment of paradoxical insomnia.

## Conclusions

There are various subtypes of insomnia and paradoxical insomnia is a less commonly known subtype. This case report aims to make the primary care providers aware of paradoxical insomnia. It also highlights the role of a good sleep history, Epworth sleepiness scale, and polysomnography as important diagnostic tools. Cognitive-behavioral therapy remains the most effective treatment for insomnia. This case aims to reinforce the importance of cognitive behavioral therapy as first-line therapy for insomnia.

## References

[1] Bhaskar S, Hemavathy D, Prasad S (2016). Prevalence of chronic insomnia in adult patients and its correlation with medical comorbidities. J Family Med Prim Care.

[2] Geyer JD, Lichstein KL, Ruiter ME, Ward LC, Carney PR, Dillard SC (2011). Sleep education for paradoxical insomnia. Behav Sleep Med.

[3] Kaufmann CN, Spira AP, Alexander GC, Rutkow L, Mojtabai R (2016). Trends in prescribing of sedative-hypnotic medications in the USA: 1993-2010. Pharmacoepidemiol Drug Saf.

[4] Sateia MJ (2014). International classification of sleep disorders-third edition: highlights and modifications. Chest.

[5] Harvey AG, Tang NK (2012). (Mis)perception of sleep in insomnia: a puzzle and a resolution. Psychol Bull.

[6] Johns MW (1991). A new method for measuring daytime sleepiness: the Epworth sleepiness scale. Sleep.

[7] Mendelson WB (1995). Effects of flurazepam and zolpidem on the perception of sleep in insomniacs. Sleep.

